# Impacts of increased temperatures on floral rewards and pollinator interactions: a meta-analysis

**DOI:** 10.3389/fpls.2024.1448070

**Published:** 2024-11-08

**Authors:** Shirley Alquichire-Rojas, Víctor M. Escobedo, Marcia González-Teuber

**Affiliations:** ^1^ Facultad de Ciencias, Universidad Católica de la Santísima Concepción, Concepción, Chile; ^2^ Dirección de Investigación, Vicerrectoría Académica, Universidad de Talca, Talca, Chile; ^3^ Centro de Ecología Integrativa, Instituto de Ciencias Biológicas, Universidad de Talca, Talca, Chile; ^4^ Facultad de Ciencias Biológicas, Pontificia Universidad Católica de Chile, Santiago, Chile

**Keywords:** climate change, warming, floral rewards, insect pollinators, nectar, pollen

## Abstract

Flowering plants produce pollinator rewards such as nectar and pollen, whose quantity and quality usually depend on the whole-plant state under specific environmental conditions. Increasing aridity and temperature linked to climate change may force plants to allocate fewer resources to these traits, potentially disrupting plant-pollinator interactions. In this study, for the first time, both quantitative review (vote-counting procedure) and meta-analytic approach were used to assess the implications of increased temperatures linked to global warming on floral rewards, including nectar (sugar concentration, content, and volume) and pollen (germination and viability), as well as on pollinator visits. Furthermore, we explored whether observed effects of warming are related either to temperature range, plant type (wild vs crop), or study approach (greenhouse vs field experiments). We also assessed the correlations between elevated temperatures and the characteristics that were affected by the temperature range. The results of the vote-counting technique showed that higher temperatures led to a decrease in floral rewards but did not affect the number of pollinator visits. Concurrently, meta-analysis detected adverse effects of warming on pollen germination and viability. Warming effects depended on the plant type for pollen germination and viability, on study approach for nectar sugar concentration and pollen germination, and on temperature range for pollen germination and pollinator visits. Additionally, we found that pollen germination and pollinator visits significantly decreased as temperature range increased. Our results showed that global warming affects floral rewards in both wild and crop plants, providing insights into the effects of changing climatic conditions on plant-pollinator interactions and pollination services.

## Introduction

Global surface temperatures have increased by approximately 1.5°C over the last two decades, threatening ecosystem functioning and biodiversity worldwide (IPCC, 2021). Global warming is expected to alter biodiversity and species distributions and disrupt ecological networks, including plant-insect interactions (Parmesan and Yohe, 2003; Kearns et al., 1998; Chen et al., 2011; González-Teuber et al., 2023). Since most flowering plants depend on pollinator assistance for seed set and reproduction, insect pollination emerges as a crucial service for the proper functioning of ecosystems (Ollerton et al., 2011; Brzosko et al., 2021; Sidemo-Holm et al., 2021). Increased temperatures may disrupt this mutualism by modifying floral traits such as morphology, scent, and rewards, affecting pollinator attraction, visits, and behavior (Dormont et al., 2019; Kuppler et al., 2016). Disruption of pollination is likely to have a global impact on the reproductive success of 90% of wild plants and the yields of 85% of major food crops (Klein et al., 2007). While several studies have independently evaluated the impact of increased temperature on the floral traits of some species (see **Table 1**), there is currently a gap in straightforward and comprehensive multi-species assessments. Conducting such multi-species tests to assess effects of warming on distinct floral traits and pollinator visits can provide valuable insight for more accurate predictions regarding the potential consequences of forecasted climate changes on plant-pollinator interactions, as well as ecosystem pollination service.

**Table 1:** Studies used in meta-analysis of response by pollinator attractors and pollinator visits to increasing air temperature including natural plant populations and crops, response trend and number of observations reported in each publication.

Pollinator attractors	Plant type	Effect	No. of observations	Reference
Nectar sugar concentration	Natural plant populations	↓	2	Descamps et al. (2021b)
		↓	2	Descamps et al. (2021c)
		↕	1	Göttlinger and Lohaus (2019)
		↓	2	Descamps et al. (2018)
		↓	3	Takkis et al. (2015)
		↕	2	Carrión-Tacuri et al. (2012)
	Crops	↓	4	Descamps et al. (2020)
		↕	2	Muniz et al. (2013)
	Percentage of cases supporting the model	63		
Nectar sugar content	Natural plant populations	↕	12	López-Atanacio et al. (2022)
		↑	1	Maluf et al. (2022)
		↓	2	Descamps et al. (2021a)
		↕	2	Descamps et al. (2021b)
		↑	2	Descamps et al. (2021c)
		↕	2	Descamps et al. (2018)
		↕	2	Mu et al. (2015)
		↑	3	Takkis et al. (2015)
		↑	2	Carrión-Tacuri et al. (2012)
	Crops	↑	12	Clearwater et al. (2021)
		↓	4	Descamps et al. (2020)
		↓	2	Enkegaard et al. (2016)
		↑	2	Muniz et al. (2013)
		↕	2	Hoover et al. (2012)
	Percentage of cases supporting the model	21		
Nectar volume	Natural plant populations	↓	3	Moss and Evans (2022)
		↓	2	Descamps et al. (2021a)
		↓	2	Descamps et al. (2021b)
		↓	2	Descamps et al. (2021c)
		↓	2	Descamps et al. (2018)
		↓	2	Mu et al. (2015)
		↓	6	Takkis et al. (2015)
		↕	2	Carrión-Tacuri et al. (2012)
		↓	14	Mačukanović-Jocić et al. (2004)
	Crops	↓	4	Descamps et al. (2020)
		↓	2	Enkegaard et al. (2016)
		↓	2	Muniz et al. (2013)
		↕	1	Hoover et al. (2012)
	Percentage of cases supporting the model	84		
Pollen germination	Natural plant populations	↓	3	Branch and Sage (2018)
	Crops	↓	2	Rutley et al. (2021)
		↓	18	Bhandari et al. (2020)
		↓	2	Parrotta et al. (2016)
		↕	8	Li et al. (2015)
		↓	8	Lora et al. (2012)
		↓	4	Müller et al. (2016)
	Percentage of cases supporting the model	85		
Pollen viability	Natural plant populations	↓	3	Branch and Sage (2018)
		↓	2	Descamps et al. (2018)
	Crops	↓	2	Iovane and Aronne (2022)
		↓	13	Xu et al. (2017)
	Percentage of cases supporting the model	100		
Pollinator visit	Natural plant populations	↑	6	López-Atanacio et al. (2022)
		↑	3	Maluf et al. (2022)
		↓	2	Moss and Evans (2022)
		↕	4	Creux et al. (2021)
		↓	1	Descamps et al. (2021b)
		↓	4	Descamps et al. (2018)
		↑	3	Norgate et al. (2010)
	Crops	↕	34	Muniz et al. (2013)
	Percentage of cases supporting the model	38		

Percentage of publications supporting effects size and observed negative or positive trend for pollinator attractors traits and pollinator visits under increasing air temperature treatments. (↑) significative increasing effect, (↓) significative decreasing effect, (**↕**) effect remained constant.

Floral rewards such as nectar and pollen play a major role in the acceptance of the flower by pollinators, serving as primary food sources for them (Hegland et al., 2009; Willmer, 2011; Celedón-Neghme et al., 2016; Vaudo et al., 2020, 2024). Nectar is rich in carbohydrates and amino acids (González-Teuber and Heil, 2009a, 2009b), whereas pollen provides essential proteins and lipids (Roulston and Cane, 2000). Higher concentrations of these metabolites usually enhance floral attractiveness of flowers to visiting insects (Somme et al., 2015; Zhao et al., 2016; Descamps et al., 2018). Increased temperatures have been linked to alterations in nectar and pollen (Borghi et al., 2019). Some studies have reported that both nectar volume and sugar content and concentration are negatively impacted by increased temperatures (Descamps et al., 2018, 2020, 2021a, 2021b), although effects appear to be species-dependent (Descamps et al., 2020; Göttlinger and Lohaus, 2019). Similarly, pollen fertilization traits appear to be highly sensitive to elevated temperatures (Raja et al., 2019). Many studies have shown that increased temperatures often lead to pollen abortion and asynchronous pollen and stigma development, negatively affecting pollen viability, germination, and pollen tube growth (Parrotta et al., 2016; Müller and Rieu, 2016). While variations in pollen quality (i.e., nutritional composition) in response to increased temperatures have been little studied, some studies have shown evidence that reductions in protein and starch concentrations contribute to failures in pollen development and viability (Pressman et al., 2002; Sato et al., 2006; Muth et al., 2016), suggesting a link between pollen nutrition and pollen fertilization.

Floral trait modifications may lead to bottom-up effects on flower visitation by pollinators, affecting their foraging behavior, pollen transfer, and, ultimately, plant reproductive success. Although pollinator flower preferences are unequivocally based on nectar and pollen taste, how variations in these floral rewards may alter pollinator behavior in a warming climate remains uncertain (Descamps et al., 2018, 2021a, 2021b). Here, we assembled a global dataset of distinct floral traits (nectar volume, nectar sugar concentration, nectar sugar content, pollen germination, and pollen viability) and pollinator visitation to test how these traits change under increased temperatures in a global warming context. We provide a comprehensive quantitative synthesis and conduct a meta-analysis based on available data in the published literature, examining the effects of experimentally increased temperature on multi-trait displays of flowers in both natural plant populations and crop species. Since floral traits attract pollinators, we hypothesize that reductions in floral rewards (nectar and pollen) linked to climate warming should be accompanied by decreased pollinator visits. Specifically, we initially tested, using the available literature, whether experimentally increased temperatures harm floral rewards and pollinator visits. Subsequently, we assessed how the increase in air temperature influences floral rewards and pollinator visits, considering various explanatory variables (plant type, study approach, and temperature range). Furthermore, we explored relationships between increased air temperature and all evaluated traits impacted by global warming. Relevant implications for pollinator services and recommendations for future research directions are additionally discussed. While there is evidence of floral trait responses to climate change drivers such as drought (see Kuppler and Kotowska, 2021; Jaworski et al., 2022), to our knowledge, this study represents the first direct, comprehensive assessment of the potential effects of global warming on floral rewards and floral-visitor interactions.

## Materials and methods

### Study selection and data collection

We conducted a search in the Web of Science and Scopus using multiple search term combinations with no restriction on publication years for one of the following flower rewards traits keywords: nectar sugar concentration ‘OR’ nectar sugar content ‘OR’ nectar volume ‘OR’ pollen viability ‘OR’ pollen germination ‘OR’ pollinator visit ‘OR’ plant-pollinator interaction; AND all of following global warming keywords: climate change ‘OR’ global warming ‘OR’ heat stress ‘OR’ high air temperature. Our initial search yielded 1561 publications, which were reviewed to assess their suitability. To be included in the analysis, studies had to inform measurements of floral rewards and/or pollinator visits (i.e., mean value, standard deviation or standard error, and sample size) in control and observational or experimental air temperature increase treatments. To ensure the comparability of studies, we established a maximum range of increased temperature treatment of 12°C. Despite both relating to nectar quality, we distinguished nectar sugar concentration and nectar sugar content. The former refers to amount per gram of tissue, while the latter exclusively refers to amount per tissue (Brzosko et al., 2021; Descamps et al., 2021a; Nicolson, 2022). Studies regarding variations in the nutritional composition of pollen as a consequence of increased temperatures could not be included in the meta-analysis, because of the low number of available studies (Egger et al., 1997). A total of 28 publications met these criteria, and we estimated the percentage of studies verifying the effect of increased air temperature on floral reward traits and pollinator visits (vote-counting procedure; **Table 1**).

### Statistical analysis

To examine the mean effects of experimentally increased temperature (warming) on floral traits and pollinator visits, we calculated Hedge’s effect size (*g*) using the *scalc* function from the *metafor* library (Viechtbauer, 2010) in R environment (R Core Team, 2024). Hedge’s *g* represents the standardized difference in means between floral traits (and pollinator visits) under increased and non-increased or control temperature conditions. Positive values of *g* indicate an increase in floral reward traits (or pollinator visit) following warming, whereas negative values signify a decrease. We conducted a random-effects meta-analysis using the *rma.mv* function for each floral trait, incorporating “species” and “study” as random factors. The former was included to account for variability across plant responses to temperature increases, and the latter to address heterogeneity among study cases (Gurevitch and Hedges, 1999):

M1<–*rma.mv(EffectSize*, *Vi*, *random* = *list*(~*1*|*study*, ~1|*species*)*, data = Data*)

Heterogeneity tests (Q_T_ and Q_M_) were performed to assess effect size homogeneity in each of the six analyses (nectar sugar concentration, nectar sugar content, nectar volume, pollen germination, pollen viability, and pollinator visit). All analyses showed significant heterogeneity (**Table 2**). To examine sources of variation, we included explanatory variables (i.e., moderators): plant type (wild vs crop), study approach (greenhouse vs field experiments), and increased temperature ranges ([0.99; 4°C], [4.1; 8°C], and [8.1; 12°C]). Categorical random-effects models tested the effects of each moderator on floral reward traits and pollinator visits.

**Table 2:** Total heterogeneity (Q_T_) and between-group heterogeneity (Q_M_) of man effect sizes (Hedges’ *g*) in studies comparing floral reward traits in response to increasing air temperature in phylogenetically uninformed and informed models.

Floral reward trait	Moderator	Phylogenetically uninformed model				Phylogenetically informed model			
		Q_T_	*p*-value	Q_M_	*p*-value	Q_T_	*p*-value	Q_M_	*p*-value
Nectar sugar concentration	Overall	86.4	**<0.001**			86.4	**<0.001**		
	Plant type			1.24	0.536			4.19	0.123
	Study approach			8.82	**0.012**			5.87	0.053
	Temperature range			2.39	0.496			2.23	0.524
Nectar sugar content	Overall	237	**<0.001**			237	**<0.001**		
	Plant type			0.46	0.796			0.32	0.849
	Study approach			1.04	0.592			1.02	0.600
	Temperature range			0.52	0.913			0.50	0.917
Nectar volume	Overall	277	**<0.001**			277	**<0.001**		
	Plant type			3.17	0.204			1.4	0.495
	Study approach			3.58	0.167			1.95	0.376
	Temperature range			4.04	0.257			2.14	0.544
Pollen germination	Overall	599	**<0.001**			599	**<0.001**		
	Plant type			32.9	**<0.001**			12.8	**0.002**
	Study approach			27.3	**<0.001**			10.28	**0.006**
	Temperature range			31.4	**<0.001**			17.5	**0.001**
Pollen viability	Overall	158	**<0.001**			162	**<0.001**		
	Plant type			5.78	**0.055**			5.76	**0.056**
	Study approach			–	–			–	–
	Temperature range			5.21	0.074			5.36	0.147
Pollinator visits	Overall	608	**<0.001**			576	**<0.001**		
	Plant type			1.13	0.567			1.72	0.421
	Study approach			1.14	0.535			0.48	0.785
	Temperature range			44	**<0.001**			56.2	**<0.001**

Q_T_ values are given for models without data structure (i.e. no moderator). Significant p-values (<0.05) of Q_T_ and Q_M_ are shown in bold.

Additionally, we carried out linear meta-analysis random models to evaluate the association between the reported increased temperature (as a continuous moderator) and warming-influenced traits. Models included studies as a random factor again, and heterogeneity tests were also implemented. Despite the small number of plant species included in the analysis, we implemented a phylogenetic correction for the overall model by specifying a species phylogenetic correlation matrix in the R argument of the *rma.mv* function. We constructed a phylogenetic tree of the species in this study based on the megaphylogeny of plants (Jin and Qian, 2023) using the *S.Phylomaker* function (Jin and Qian, 2022) and *phytools* package (Revell, 2012). Then, we extracted the correlation matrix for all species using the *vcv* function from the *ape* package (Paradis and Schliep, 2019), which applies a Brownian-motion evolution model. In this matrix, closely related species exhibit higher correlations, reflecting their expected similarities. The Egger et al. (1997) test was used to detect publication bias, and a weighted method was applied to calculate the fail-safe number to evaluate whether unpublished data may have affected our conclusions (Rosenberg, 2005).

## Results

**Figure 1:**
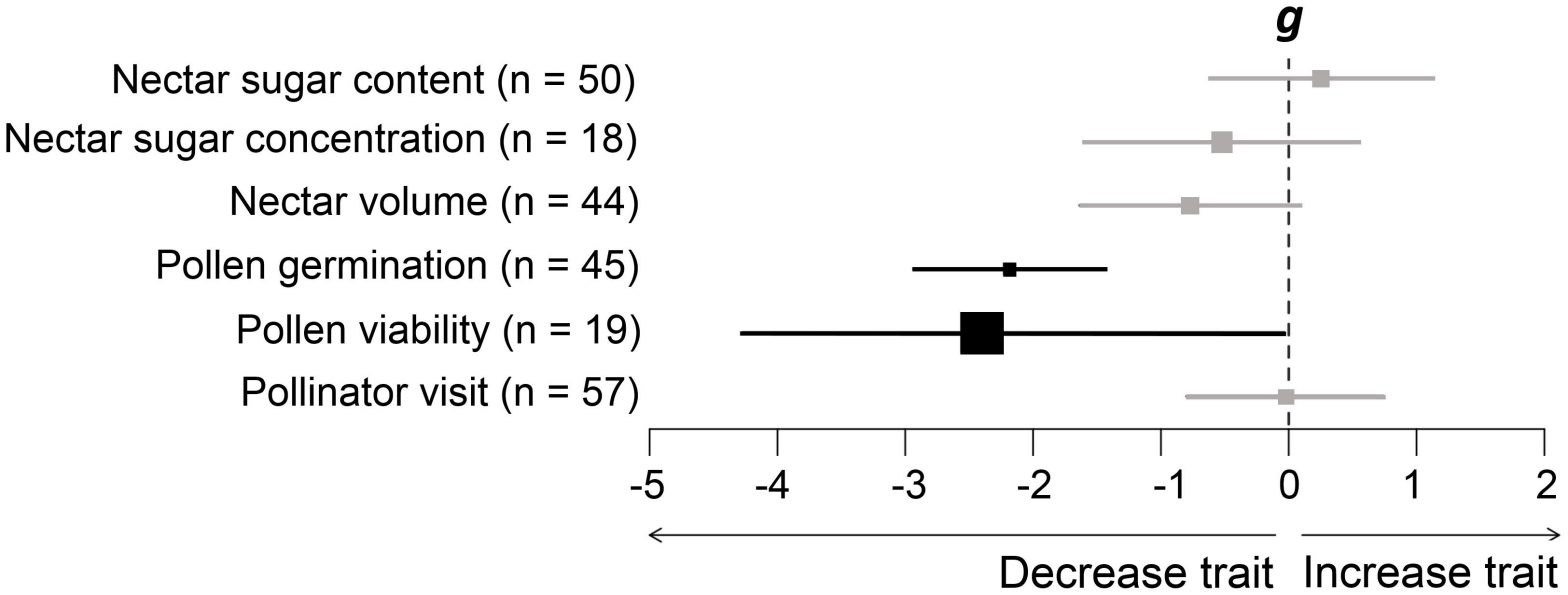
Mean effect sizes (Hedges’ *g*) of differences in floral reward traits in response to increasing air temperature in phylogenetically uninformed models. Error bars depict 95% confidence intervals (CIs). A mean effect size is significantly different from zero when CIs do not overlap zero. Significant results are shown in black. Negative (or positive) effect sizes indicate a decrease (or increase) in floral reward traits due to warming.

The literature survey (i.e., vote-counting) found that warming (increased temperature) had detrimental effects on floral reward traits and pollinator visits (65% of cases) (**Table 1**), considering 31 plant species (**Supplementary Table S1**). Information about origin of plants species is indicated in **Supplementary Table S1**. Specifically, warming decreased nectar sugar concentration (63% of cases), nectar sugar content (21% of cases), nectar volume (84% of cases), pollen germination (85% of cases), pollen viability (all cases), and pollinator visit (38% of cases). The meta-analyses showed that warming significantly decreased pollen germination (*g* = -2.19, *p =* 0.000) and viability (*g* = -2.41, *p =* 0.047), whereas nectar sugar concentration, nectar volume, and even pollinator visits tended to non-significantly reduce (**Figure 1**). Conversely, nectar sugar content tended to increase due to warming, though not significantly (*g* = 0.25, *p =* 0.572). These results were consistent for the phylogenetically uninformed-informed models (**Supplementary Figure S1**). We did not detect trait phylogenetic signals, which indicate that study species are less related than expected by chance, suggesting that species can be considered as independent samples. Alternatively, the lack of phylogenetic signal can be due to the small size of the phylogeny (Chamberlain et al., 2012). Phylogenetic corrections were found to result in more conservative estimators, likely influenced by the phylogenetic relationships among species, despite the inclusion of 16 distinct plant families in the study.

**Figure 2:**
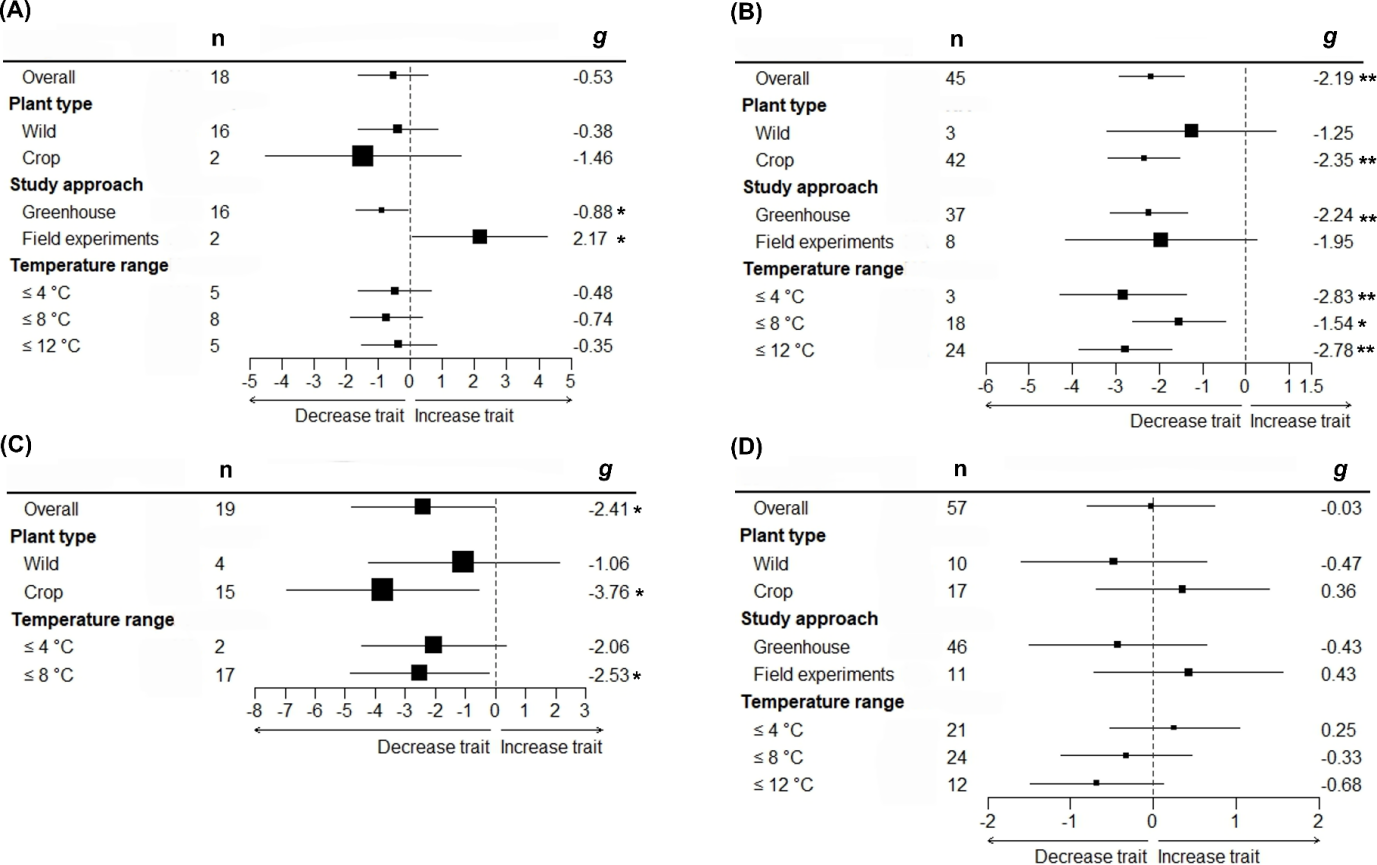
Group-specific mean effect sizes (Hedges’ *g*) for floral rewards traits in response to increasing air temperature. Nectar sugar concentration **(A)**, Pollen germination **(B)**, pollen viability **(C)** and pollinator visit **(D)** phylogenetically uninformed models. Effect size *g* have been grouped according to plant type, study approach and temperature range. Mean effect size, their 95% confidence interval (CI) and the number of effect sizes (n) for phylogenetically uninformed overall and with moderator models are shown. *P < 0.05; **P < 0.01.

**Figure 3:**
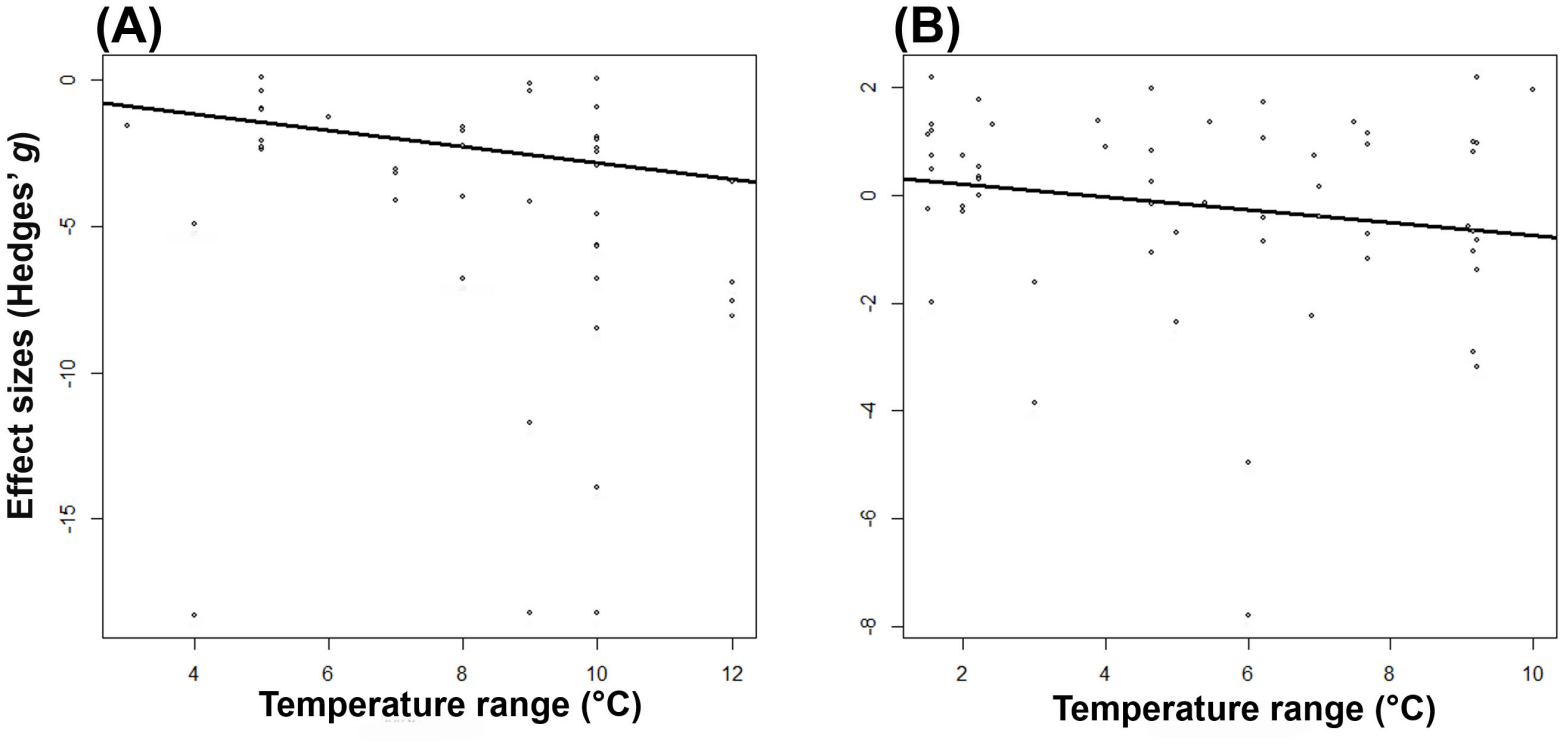
The relationship between effect sizes (Hedges’ *g*) and increased air temperature of pollen germination (P < 0.05) **(A)** and pollinator visit (P < 0.05) **(B)**.

We found that for only some studied traits moderators explained a significant proportion of their variation in Hedges’ effect sizes (*g*) (**Table 2** and **Figure 2**). While effect sizes of nectar volume and sugar content did not differ significantly by any moderator, we found a significant effect of between-group heterogeneity (Q_Ms_) for nectar sugar concentration, pollen germination, pollen viability, and pollinator visits (**Table 2**). Specifically, nectar sugar concentration was influenced by study approach moderator, showing a significant decrease and increase in *g* values under greenhouse and field experiments, respectively (**Figure 2A**). Pollen germination and viability were influenced by temperature range moderator, where *g* values in crop species consistently decreased, but in wild plant type, they did not change (**Figures 2B, C**). Although *g* values in pollinator visits are being influenced by temperature range moderator (**Table 2**), no significant decrease was observed for any temperature range category (**Figure 2D**). The *g* values for pollen germination (*z* = -4.470, *p* = < 0.05) and pollinator visits (*z* = -5.976, *p* = < 0.05) were observed to significantly decrease as experimental temperature increases (**Figures 3A, B**).

## Discussion

This study represents the first comprehensive, multi-species examination of the impact of global warming (i.e., increased temperature experiments) on floral rewards, encompassing nectar and pollen-associated traits, and plant-pollinator interactions. A quantitative literature survey, including 31 plant species from tropical and temperate climates, supported detrimental effects of increased temperature on floral reward traits and pollinator visits in 65% of cases. Our meta-analysis revealed that pollen-associated traits were highly sensitive to increased temperature, whereas negative yet non-significant effects of increased temperature were reported on nectar traits (except nectar sugar content) and pollinator visits. Significant heterogeneity in effect size was detected across all analyses, with the primary explanatory factor being the experimental temperature range, followed by plant type (wild vs crop), and lastly, study approach (greenhouse vs field experiment).

Contrary to our expectations, nectar rewards (i.e., nectar sugar content, nectar sugar concentration, and nectar volume) showed no consistent pattern in response to temperature treatments. Significant heterogeneity in effect size, however, was observed for nectar sugar concentration; some of which was explained by the study approach (greenhouse vs field experiments). Nectar sugar concentration showed a significant inverse tendency depending on this moderator, declining in response to temperature increases under controlled conditions, while increasing under field conditions. An earlier meta-analysis of floral trait responses showed similar divergence between controlled- and field experiments, with declines in nectar volume in response to water deficit observed in indoor conditions, but not outdoor conditions (Kuppler and Kotowska, 2021). Even when mechanisms involved in these opposite tendencies between indoor and outdoor conditions are not fully understood, some factors might help to explain this phenomenon. For example, in natural conditions (i.e., field experiments) plants are compelled to invest in nectar production to maintain pollinator interactions, despite potential costs it may involve in stressful environments (Willmer, 2011). This investment is likely unnecessary in indoor conditions in the case that no pollinators consume the nectar. Nectar is a plastic trait, whose secretion may be adjusted according to the intensity of consumption, or, it can be even reabsorbed in the case of non-consumption (Pacini et al., 2003). Furthermore, nectar volume and concentration are highly influenced by environmental factors such as air temperature and humidity. Many of the studies, covered in this meta-analysis, quantified either the nectar volume or its concentration, but not both. The latter might lead to high variations in nectar secretion measurements, since both must be considered together in order to effectively calculate the realistic amounts of secreted soluble solids (Heil, 2011). While nectar variation might be an inherent limitation across studies, the use of a rigorous statistical meta-analytic framework has allowed us to have a reliable estimation of the effects of increased temperatures on nectar traits.

Our results supported the detrimental effects of increased temperature on both pollen germination and viability; nevertheless, declines in pollen germination and viability were only observed for crop species, but not for wild species (**Figure 2**). Numerous studies have shown that crops are highly sensitive to temperature increases, particularly during reproductive phases (Driedonks et al., 2016). Crop plants are more sensitive to abiotic stresses than their wild-type relatives because breeding selection for yield is not necessarily linked to an adaptive stress tolerance strategy (Villalobos-López et al., 2022).

Pollen germination and viability usually have a direct influence on seed set and fruit set ratio. Even when impacts of increased temperatures on seed and/or fruit set were not included in this meta-analysis, numerous studies have reported that negative effects on pollen viability and germination due to higher temperatures are accompanied by declines in seed set (Powell et al., 2012; Tolessa and Heuvelink, 2018; Yang et al., 2019; Shenoda et al., 2021). Given that 75% of all food crop species rely on insect pollination for seed set (Klein et al., 2007), higher temperatures potentially represent a threat to yields and food security. Pollen germination and viability were significantly reduced by temperature range (**Figures 2B, C**; **3A**), with the former more sensitive to temperature increases than the latter. Whereas pollen germination was negatively affected by all temperature increments (**Figure 2B**), pollen viability was only negatively affected by temperatures over 8°C (**Figure 2C**). Development and functioning of the male gametophyte (or pollen) are known to be the most temperature-sensitive processes within the plant life cycle (Zinn et al., 2010). Furthermore, both pollen germination and viability are highly sensitive at even short periods of high temperature exposure (Müller et al., 2016). Thus, differences observed in the temperature-sensitive range between pollen germination and viability might be explained by genetic variation for thermotolerance among plant species included in the current study.

The results from our meta-analysis and literature survey did not align with the prediction of detrimental effects on pollinator visits due to warming. Specifically, the meta-analysis showed that temperature increases did not significantly affect pollinator visits. Likewise, the literature survey only recorded 38% support for our prediction. Although the high heterogeneity of effect sizes could certainly be a contributing factor, results might be at least partially explained by the temperature range (**Table 1**; **Figure 3B**). There was a clear trend of decreasing pollinator visitation with increasing temperature (**Figure 3B**). It is known that elevated temperatures can influence pollinator activity both directly and indirectly (Descamps et al., 2018). Whereas direct effects are mainly associated with changes in pollinator activity and flower-visiting behavior (Descamps et al., 2018), indirect ones (i.e., those mediated via at least one other interacting species) may result from changes in floral signals and rewards (Knauer and Schiestl, 2015; Descamps et al., 2018). Insect pollinators are typically only active within certain temperature limits, becoming inactive at temperatures above and below these thresholds (Corbet et al., 1993; Kühsel and Blüthgen, 2015; Plos et al., 2023). Moreover, since pollinators rely exclusively on floral rewards for food (Hegland et al., 2009; Willmer, 2011), variations in the quantity and quality of nectar and pollen are expected to lead to changes in pollinator-visiting behavior; and thereby, may influence plant reproduction (Robertson et al., 1999; Konzmann and Lunau, 2014; Descamps et al., 2018; Plos et al., 2023). In our study, temperature effects on pollinator visits were not differentiated based on either direct or indirect effects (information not provided in the studies). Since both types of effects may interact (Kharouba et al., 2018; Kharouba and Yang, 2021; Freimuth et al., 2022), future research should consider both in order to determine the overall outcome of climate change impacts on species interactions.

Our meta-analysis revealed that increased temperatures affect floral rewards, particularly pollen traits, which likely scale up to pollinator interactions. Climate models predict that the global mean temperature will increase by 1-4°C by the end of the twenty-first century (IPCC, 2021). Nevertheless, most detrimental effects on plants seem to be caused by heat waves or extreme temperature events (López et al., 2022), which are projected to increase in both intensity and frequency (Meehl et al., 2007). Heat waves are predicted to have short-term durations of a few days, but with an increase in temperatures of over 5°C (IPCC, 2021). Heat-induced pollen damage is related to reductions in seed and fruit set. Therefore, understanding how floral traits, particularly pollen development, respond to extreme weather events is key to predicting how reproduction in natural plant populations and agricultural systems may be affected by climate change. Moreover, if heat-related shifts in pollen traits are likely associated with a decline in pollinator visits, this scenario may be much more serious. A recent meta-analysis reported that without pollinators, half of all the flowering plants would suffer a decline in fertility of over 80%, while a third would not produce seeds at all (Rodger et al., 2021). While ecological consequences of other climate change-associated factors such as drought on floral traits and plant-pollinator interactions are relatively well established, to our knowledge, this is the first meta-analytic approach to assess potential effects of global warming on floral rewards and floral-visitor interactions. Elevated temperatures, however, are usually accompanied by increased risk of a range of other abiotic stresses (such as drought and light intensity); therefore, future studies should explore combinations of these factors to improve understanding of climate change effects on floral metabolism and plant-pollinator interactions.

## Data Availability

The raw data supporting the conclusions of this article will be made available by the authors, without undue reservation.
