# Supplementary material for: Impacts of increased temperatures on floral rewards and pollinator interactions: a meta-analysis
Source: Front Plant Sci. 2024 Nov 8;15:1448070. doi: 10.3389/fpls.2024.1448070 (PMC11581868; doi:10.3389/fpls.2024.1448070)
Supplement: Supplementary file 1 [file Presentation1.pdf]

Supplementary Material

Impacts of increased temperatures on floral rewards and pollinator interactions: a meta-analysis

Shirley Alquichire-Rojas[1], Víctor M. Escobedo[2,3], Marcia González-Teuber[4]

[1]Facultad de Ciencias, Universidad Católica de la Santísima Concepción, Concepción, Chile

[2]Dirección de Investigación, Vicerrectoría Académica, Universidad de Talca, Talca, Chile

[3]Centro de Ecología Integrativa, Instituto de Ciencias Biológicas, Universidad de Talca, Talca, Chile

[4]Facultad de Ciencias Biológicas, Pontificia Universidad Católica de Chile, Santiago, Chile

Corresponding author: mgonzat@uc.cl

1 Supplementary Figures and Tables

Figure S1. Mean effect sizes (Hedges' *g*) of differences in floral reward traits in response to increasing air temperature in phylogenetically informed models. Error bars depict 95% confidence intervals (CIs). A mean effect size is significantly different from zero when CIs do not overlap zero. Significant results are shown in black. Negative (or positive) effect sizes indicate a decrease (or increase) in floral reward traits due to warming.

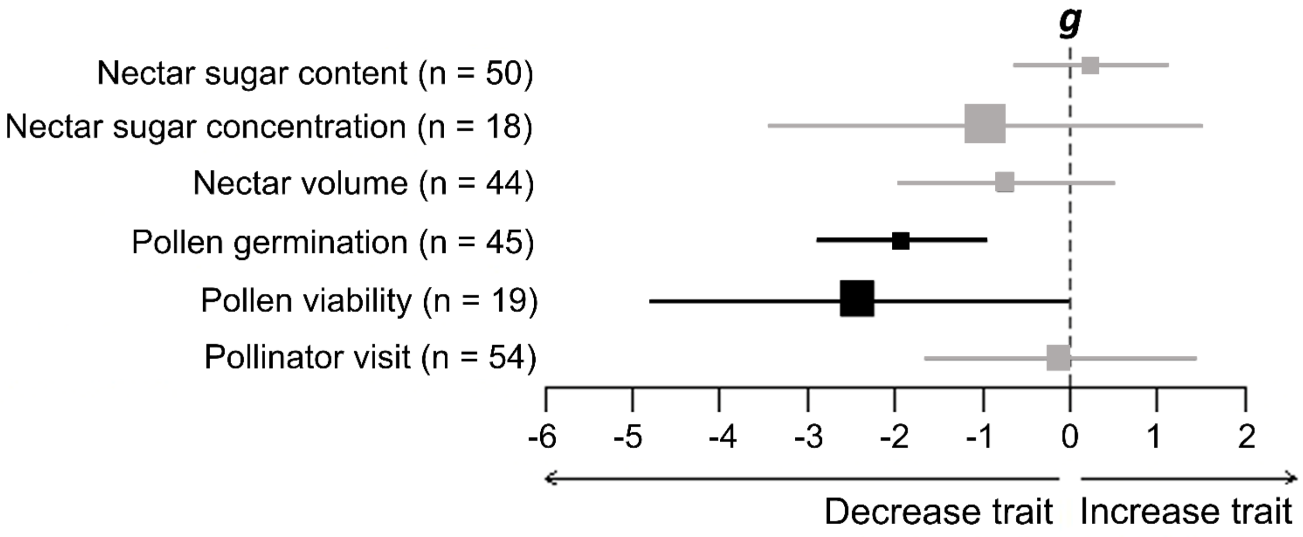

Table S1. Information regarding the origin of all plant species covered in this study. The information was facilitated by Royal Botanic Gardens, Kew in Plants of the World Online, published on the Internet; http://www.plantsoftheworldonline.org.

**Family**	**Species**	**Plant origin**	**Biome**
Verbenaceae	*Lantana camara*	Mexico to Tropical America	Subtropical
Boraginaceae	*Borago officinalis*	West and Central Mediterranean	Temperate
Balsaminaceae	*Impatiens glandulifera* Royle	North Pakistan to Nepal	Temperate
Bromeliaceae	*Aechmea fasciata*	Brazil (Rio de Janeiro)	Wet tropical
Lamiaceae	*Ballota acetabulosa*	South and East Greece to West Türkiye	Subtropical
Lamiaceae	*Teucrium divaricatum*	East Mediterranean	Subtropical
Myrtaceae	*Leptospermum scoparium*	Southeast Australia, New Zealand, Chatham Islands	Subtropical
Boraginaceae	*Echium plantagineum*	Great Britain, Macaronesia to Caucasus	Subtropical
Boraginaceae	*Echium vulgare*	Europe to Xinjiang	Temperate
Brassicaceae	*Brassica napus*	South Europe	Temperate
Lamiaceae	*Lamium galeobdolon*	Europe to Iran	Temperate
Lamiaceae	*Lamium maculatum*	Europe to North China	Temperate
Lamiaceae	*Ajuga reptans*	Europe to North Iran, Northwest Africa	Temperate
Lamiaceae	*Ocimum basilicum*	Tropical and Subtropical Asia to North Australia	Dry tropical
Solanaceae	*Solanum lycopersicum*	Peru	Wet tropical
Aizoaceae	*Trianthema portulacastrum*	Tropics and Subtropics	Dry tropical
Poaceae	*Oryza sativa*	China	Temperate
Solanaceae	*Nicotiana tabacum*	Bolivia	Dry tropical
Annonaceae	*Annona cherimola*	Southwest America	Wet tropical
Leguminosae	*Cicer arietinum*	Southeast Türkiye to Iran	Temperate
Fabaceae	*Vicia lens*	West Mediterranean, Iraq to W. Himalaya	Temperate
Asteraceae	*Centaurea cyanus*	Central and East Mediterranean	Temperate
Cucurbitaceae	*Cucurbita maxima* Duchesne	Bolivia to North Argentina	Subtropical

Cucurbitaceae	*Cucurbita melopepo*	Central and Southeast U.S.A. to Northeast Mexico	Subtropical
Asteraceae	*Glebionis segetum*	South and East Mediterranean	Temperate
Asteraceae	*Helianthus annuus*	Southwest U.S.A. to Mexico	Temperate
Asteraceae	*Saussurea nigrescens*	Qinghai to China	Temperate
Fabaceae	*Stylosanthes capitata*	South Tropical America	Dry tropical
Plantaginaceae	*Veronica persica*	Caucasus to North Iran	Temperate
Lamiaceae	*Anisomeles indica*	Tropical and Subtropical Asia	Wet tropical
Lamiaceae	*Lamiopsis purpurea*	Macaronesia, Mediterranean, Europe to West Siberia	Temperate
